# Assessment of partograph utilization and associated factors among obstetric care givers at public health institutions in central zone, Tigray, Ethiopia

**DOI:** 10.1186/s13104-018-3814-7

**Published:** 2018-10-10

**Authors:** Tesfay Hailu, Kidane Nigus, Gebreamlak Gidey, Birhane Hailu, Yohannes Moges

**Affiliations:** Department of Midwifery, College of Health Science and Referral Hospital, Aksum University, Axum, Ethiopia

**Keywords:** Partograph, Utilization, Obstetric care givers, Ethiopia

## Abstract

**Objectives:**

Partograph is one of the best effective obstetric tools used to monitoring labor and prevent prolonged or obstructed labor which accounts for about 22% of maternal deaths in Ethiopia. This study was aimed to assess partograph utilization and associated factors among obstetric care givers. Facility based cross sectional study was used in the randomly selected health facilities. Total 220 obstetric care givers were selected using simple random sampling technique. Data were entered and analyzed using SPSS version 22.0. Bivariate and multivariate logistic regression analysis was used to identify the associations of each explanatory variable with the outcome variable. Finally, odds ratio with its 95% confidence interval and p-value of 0.05 was used to identify significant variables.

**Result:**

Out of 198 obstetric care providers, 73.3% used partograph to monitor progress of labor. Those who were diploma holders (AOR = 3.8, CI = 2.2–6.2), receiving basic emergency obstetrics and new born care training (AOR = 5.6, CI 1.1–28.5), age between 20 and 29 years-old (AOR = 0.1, CI = 0.01–0.50), and male health care providers (AOR = 0.37, CI = 0.44–0.95) were factors significantly associated with partograph utilization. Partograph utilization in this study was below the WHO recommendation. Especial emphasizes and interventions should be given to increase partograph utilization.

**Electronic supplementary material:**

The online version of this article (10.1186/s13104-018-3814-7) contains supplementary material, which is available to authorized users.

## Introduction

The tragedy of maternal mortality in Ethiopia is that despite the recognition of maternal mortality as a major public health issue, maternal mortality figures continue to rise, in spite of the apparent commitment by stakeholders. The majority of the deaths and complications could be prevented by cost-effective and affordable health interventions like the partograph [1].

The partograph is an effective tool for monitoring labor, and when used effectively, it prevents obstructed labor, which accounts for about 8% of maternal deaths worldwide. Thus it serves as an ‘early warning system’ and assists in early decision on transfer, intervention decisions in hospitals and ongoing evaluation of the effect of interventions. Partograph has been promoted by the World Health Organization as the “gold” standard for assessing progress in labor in most low resource countries like Ethiopia [1, 2].

Approximately 300,000 maternal deaths occurred globally in 2013, of which 98% occurred in the developing countries. On the average 230 women die per every 100,000 live births every year in developing countries [3].

The burden of maternal death is not uniformly distributed throughout the world. Obstetric risk is by far the highest in sub-Saharan Africa. In 2015, the MMR for sub-Saharan Africa was estimated to be nearly 546 per 100,000 live birth, three times higher than that of South Asia (182 per 100,000), eight times higher than in Latin America and the Caribbean (68 per 100,000), and more than 30 times higher than in industrialized countries (16 per 100,000) [4–6].

In Ethiopia, maternal death is still high. It is estimated 412 per 100,000 live births. However, Eighty-five percent of deaths can be prevented with cost effective interventions like partograph during labor and delivery [7].

Even thought, prolonged labor is the most common cause of death among mothers and new-born in a developing country that in turn, leads the woman to face serious complication related to obstructed labor, dehydration, exhaustion, or rupture of the uterus and infection. Obstructed labor is also other most common cause of maternal death in developing world [8].

World Health Organization recommends the universal utilization of the partograph during labor and routine use of partograph is helpful to make better decisions for the diagnosis and management of prolonged and obstructed labor [9]. Studies showed that, prevention of prolonged and obstructed labor by using partograph during labor is a key intervention in the reduction of maternal and perinatal morbidity and mortality [10].

Evidences from developing country including Ethiopia demonstrated that, the utilization of partograph is poor despite preparing the tool that is simple and inexpensive for intra partum monitoring of labor [8, 10].

In Ethiopia, since the major sources of maternal and neonatal morbidity and mortality are related to poor labor and delivery care [11]. Even though, partograph is a tool which is helpful to manage obstructed labor and prevent prolonged labor with its complications, still the level of utilizations and factors affecting to use among obstetric care providers were not yet studied in the study area. Therefore, in this study, we aimed to determine the level of partograph use and identify the factors associated with its use among obstetric caregivers in central zone of Tigray, northern Ethiopia.

## Main text

### Study area and period

This study was conducted in randomly selected public health institutions of central zone of Tigray which is located at 1028 km away from the capital city of Ethiopia, Addis Ababa and 245 km from Mekelle, which is the administrative city of the Region. The study was conducted from October 2016 to April 2017.

### Study design

Institutional based cross-sectional survey was conducted.

### Sample size

The sample size was estimated using single proportion formula by assuming 5% marginal error and 95% confidence interval (σ = 0.05) and study conducted in Addis Ababa city in which the proportion of utilization of partograph among obstetric care givers were 57.3% [8]. By adding 5% for non-respondents the final sample size was taken as 220.

### Sampling procedure

All health institutions found in central zone of Tigray were included and 37 health institutions were selected using simple random sampling technique. The total sample size of the study was distributed over each of the institute proportional to their size and the required number of study subjects was selected randomly from each selected public health institutions.

### Data collection instrument and techniques

A structured questionnaire which was developed from different literatures was used. It was prepared in English and translated to Tigrigna (local language) and translated back to English to check consistency. Ten data collectors and two supervisors were hired during the data collection period.

### Data processing and analysis

Data was entered, checked, and analyzed using SPSS version 22.0. Descriptive statistics was employed to calculate frequencies, mean and percentage. Bivariable logistic regression analysis was made using OR and 95% CI to assess the association of independent variable with the outcome separately. Based on Bivariate analysis variables that showed significant association at (p < 0.2) were entered to multivariable analysis to select Predictor variables of factors affecting partograph utilization. Finally, variables that showed significant association at (p < 0.05) were identified as independent predictors of partograph utilization.

### Ethical considerations

The Ethical approval was obtained from Institutional Review Board of College of Health Sciences, Aksum University. Communications with relevant bodies was made through a formal letter obtained from regional health. The objective and importance of the study was explained to the study participants. Data was collected after full informed written consent was obtained from participants. Confidentiality of the information and privacy is maintained.

### Socio-demographic characteristics of study participants

Out of 220 study participants, 198 (90%) properly completed questionnaires were analyzed. About 65.7% of obstetric care providers were females. Majority of the study participants, 83 (41.9%) were in the age group of 20–29 years. From all study participants, 112 (56.6%) of the healthcare providers had a diploma educational status. Regarding their profession, 139 (70.2%) were midwives followed by nurses 33 (16.7%). One hundred forty-eight (74.7%) of the obstetric care givers were working at delivery ward currently while the rest were working in antennal ward, family planning and postnatal wards. Nearly half, 96 (48.5%) of obstetric caregivers, had 5 years and above of clinical service and about 130 (65.7%) of them had trained on Basic Emergency Obstetric and Newborn care training (Table 1).

**Table 1:** Socio-demographic characteristics of obstetric care givers at public health institutions in central zone, Tigray, Ethiopia/2017

Variables, N = 198	Frequency	Percentage
Sex		
Male	68	34.3
Female	130	65.7
Age		
20–29	83	41.9
30–39	52	26.3
40–49	53	26.8
≥ 50	10	5.1
Profession		
Health officer	26	13.1
Nursing	33	16.7
Midwifery	139	70.2
Level of education		
Diploma	112	56.6
Degree	71	35.9
Others	15	7.6
Working experience		
Less than 2 years	50	25.3
2–5 years	52	26.3
5 years and above	96	48.5
Current working department		
Delivery room	148	74.7
ANC/PNC/FP	23	11.6
Other	27	13.6
Involved in labor and delivery		
Yes	192	97.0
No	6	3.0
Received training on BEmONC		
Yes	130	65.7
No	68	34.3

### Level of knowledge towards the use of partograph

Overall 135 participants (68.2%) had a satisfactory knowledge of benefit of partograph and recording observation on partograph (Additional file 1: Table S1).

### Attitude of the participant towards the use of partograph

Most of study participants, 134 (67.7%), had favorable attitude towards partograph utilization. Almost all the study participants, 197 (99.5%) agreed that using partograph decreases risks of mother/infant morbidity and mortality (Additional file 1: Table S2).

### Practice of participants towards the use of partograph

The majority, 73.3% (95% CI 67.7–79.8) of obstetric care providers were utilize partograph to monitor labor. The practice of respondents on frequency of recording observation on the partograph shows: 146 (100%) for cervical dilatation, 144 (98.6%) for fetal heartbeat, 146 (100%) for color of liquor, 146 (100%) for contraction, 142 (97.3%) for descent, 146 (100%) for maternal BP, 145 (99.3%) for maternal pulse, 146 (100%) for the first dilatation plotted on the alert line, and 139 (95.2%) for membrane intact or ruptured (Table 2).

**Table 2:** Practice of partograph among obstetric care givers at public health institutions central zone, Tigray, Ethiopia/2017

Variable, N = 198	Frequency	Percent
Health institution have labor management protocol		
Yes	183	92.4
No	15	7.6
Partograph attached to the chart and properly filled		
Yes	146	73.7
No	52	26.3
Membrane intact/ruptured recorded		
Yes	139	95.2
No	7	4.8
FHB plotted half hourly		
Yes	144	98.6
No	2	1.4
Color of liquor recorded		
Yes	146	100
No	0	0
First dilatation plotted on the alert line		
Yes	146	100
No	0	0
Cervical dilatation plotted 4 h apart		
Yes	146	100
No	0	0
Descent plotted four hourly		
Yes	142	97.3
No	4	2.7
Uterine contraction plotted half hourly		
Yes	146	100
No	0	0
Maternal BP recorded on admission		
Yes	146	100
No	0	0
Maternal pulse monitored every 30 min		
Yes	145	99.3
No	1	0.7
Mother delivered in health center		
Yes	135	92.5
No	11	7.5
According the partograph the women delivered		
On or left of alert line	104	77.0
Between alert and action line	21	15.6
At or beyond action line	10	7.4

### Factors associated with partograph utilization by obstetric care providers

According to the multivariable analysis, obstetric care providers holding a diploma were 3.8 times more likely to use the partograph than M.Sc. and above [AOR (95% CI) 3.8 (2.20–6.20)]. Obstetric caregivers who were received Basic emergency obstetric and new born care training were 5.6 times more likely to use the photograph than those who did not trained [AOR (95% CI) 5.6 (1.10–28.50)]. Moreover, caregivers whose age between 20 and 29 years-old were 90% less likely to use partograph during labor and delivery than those ages ≥ 50 years-old [AOR (95% CI) 0.1 (0.01–0.50)]. Similarly, Male obstetric caregivers were also 63% times less likely to use the partograph than their counterparts [AOR (95% CI) 0.37 (0.44–0.95)] (Table 3).

**Table 3:** Factors associated with partograph utilization of obstetric care giver in public Health institution in central zone, Tigray, Ethiopia/2017

Variables	Utilization of partograph		COR (95%CI)	AOR (95%CI)
	Yes	No		
Age				
20–29	64 (77.1%)	19 (22.9%)	0.3 (0.07–1.14)*	0.10 (0.01–0.50)*
30–39	34 (65.4%)	18 (34.6%)	0.53 (0.14–2.07)	0.22 (0.03–1.55)
40–49	43 (81.1%)	10 (18.9%)	0.23 (0.10–0.69)	0.10 (0.01–0.41)
≥ 50	5 (50%)	5 (50%)	1	1
Sex				
Male	57 (83.8%)	11 (16.2%)	0.42 (0.20–0.90)*	0.37 (0.44–0.95)*
Female	89 (68.5%)	41 (31.5%)	1	1
Profession				
Health officer	17 (65.4%)	9 (34.6%)	1.3 (0.52–3.07)	3.9 (0.61–4.70)
Nursing	31 (93.9%)	2 (6.1%)	0.15 (0.04–0.67)	0.23 (0.30–1.80)
Midwifery	98 (70.5%)	41 (29.5%)	1	
Level of education				
Degree	86 (76.8%)	26 (23.2%)	4.23 (0.53–3.73)	8.0 (0.45–4.27)
Diploma	46 (64.8%)	25 (35.2%)	7.61 (0.95–6.30)*	3.8 (2.20–6.20)*
M.Sc. and above	14 (93.3%)	1 (6.7%)	1	1
Working experience				
Less than 2 years	44 (88.0%)	6 (12.0%)	0.32 (0.12–0.82)	1.2 (0.30–5.30)
2–5 years	35 (67.3%)	17 (32.7%)	1.12 (0.54–2.32)	3.2 (0.93–11.14)
5 years and above	67 (69.8%)	29 (30.2%)	1	
Received training on BEmONC				
Yes	89 (68.5%)	41 (31.5%)	2.4 (1.13–5.02)*	5.6 (1.10–28.50)*
No	57 (83.8%)	11 (16.2%)	1	1

* p-value < 0.05

### Discussion

The aim of this study was to identify utilization of partograph and associated factors among obstetric care providers in central zone of Tigray, northern Ethiopia. The prevalence of partograph utilization was found to be 73.3%. This finding is lower than studies conducted in Delta Region of Nigeria (98.8%) and Calabar, Nigeria (80.7%) [12, 13]. But this study result was higher than the findings from other studies like; Addis Ababa, Ethiopia (57.4%) [8] and in South West Nigeria (32.3%) [14].

The possible reason for this variation could be due to the availability of well-designed and coordinated programs like; the strength of mentorship, supportive supervision. Other reasons might be time of the study, competency level and background characteristics of the study participants.

This study revealed that obstetric care givers, whose age range between 20 and 29 years-old were less likely to use partograph than their counterparts. This finding is in contrast with study done in Addis-Ababa, Sidama and Assela in which age of the participants did not show a significant association with partograph utilization [8, 15, 16]. The possible reason for this difference could be due to difference in age categorization, sample size and time of study. On the other hand it can be due to non-experienced fresh graduates may have gaps on their level of competency during the pre-service training on how to plot partograph.

Findings of the current study also indicated that significant association between sex and partograph utilization. Being female’s obstetric care givers towards partograph utilization were higher than among male. This might be due to females are closer to obstetric information as they have a tendency to become midwives which makes more likely to have good knowledge of components of the partograph to utilize it than males. This finding may be entry point for health institutions and other stake holders to work on how to increase male involvement in partograph utilization. This finding is consistent with a previous study done in Assela, Ethiopia [15].

The utilization of the partograph was significantly higher among obstetric care givers holding a diploma compared to Masters of Science and above. This is consistent with the study conducted in East Gojjam, Ethiopia. It might be due to the fact that diploma holders are assigned in the remote non equipped health centers comparing to Masters of Science and above holders and might have a better chance to get refreshment training to use the partograph in identifying abnormal labor progress early, as well as arranging for the timely referral to higher health facilities [17].

Training on basic emergency obstetrics and newborn care had a significant association with partograph utilization. Obstetric care providers who received training on basic emergency obstetrics and newborn care were about 5.6 times more likely to utilize partograph than their counterpart. This finding is consistent with a previous study done in Shoa, Ethiopia. This observation could be explained by the fact that, obstetric care providers who received training had better exposure to practice about partograph and in basic emergency obstetric and new born care training, partograph is included as an indicator [9].

### Conclusion

Although world health organization recommends utilizations of partograph for all laboring women greater than one-fourth of obstetric care givers in this study hadn’t used partograph. Age, sex, Level of education and presence of training were significantly associated with utilization of partograph.

### Recommendation

Providing obstetric care training for obstetric care givers about partograph in particular, would improve partograph utilization. Furthermore, regular supportive supervision will be important to improve obstetric care in general and proper utilization of the partograph.

To have a complete picture of the situation, involve private health care providers and increasing the sample size and employing other methods may furnish better results and complement our findings.

## Limitation of the study

Relatively small sample size which may affect estimate of a parameter and power of the test. Inclusion of private health care providers would have given comprehensive picture and make generalization possible. However, findings from this study can be regarded as a snapshot of current knowledge and practice of partograph utilization within the study area.

## Additional file

**Additional file 1: Table S1.** Level of Knowledge of Partograph among obstetric care givers at public health institutions in central zone, Tigray, Ethiopia/2017. **Table S2.** Attitude of the participant towards the use of partograph among obstetric care givers at public health institutions in central zone, Tigray, Ethiopia/2017.

## Abbreviations

CI: confidence interval
AOR: adjusted odd ratio
SPSS: Statistical Package for Social Sciences
EDHS: Ethiopian Demographic and Health Survey
M.Sc.: Master of Science
BSc: Bachelor of Science
TRHB: Tigray Regional Health Bureau
MMR: maternal mortality rate
BEmONC: basic emergency obstetrics and newborn care
