# Supplementary material for: Assessment of partograph utilization and associated factors among obstetric care givers at public health institutions in central zone, Tigray, Ethiopia
Source: BMC Res Notes. 2018 Oct 10;11:710. doi: 10.1186/s13104-018-3814-7 (PMC6180516; doi:10.1186/s13104-018-3814-7)
Supplement: Supplementary file 1 — Additional file 1: Table S1. Level of Knowledge of Partograph among obstetric care givers at public health institutions in central zone, Tigray, Ethiopia/2017. Table S2. Attitude of the participant towards the use of partograph among obstetric care givers at public health institutions in central zone, Tigray, Ethiopia/2017. [file 13104_2018_3814_MOESM1_ESM.docx]

**Additional file:**

**Table S1: Level of Knowledge of Partograph among obstetric care givers at public health institutions in Central zone, Tigray, Ethiopia/2017**

| **Variables, N = 198** | **Frequency** | **Percentage** |
| --- | --- | --- |
| **Benefit of Partograph** |  |  |
| To diagnose prolonged labor |  |  |
| yes | 180 | 90.9 |
| No | 18 | 9.1 |
| To diagnose fetal distress |  |  |
| yes | 165 | 83.3 |
| No | 33 | 16.7 |
| Helps for early action or referral |  |  |
| Yes | 164 | 82.8 |
| No | 34 | 17.2 |
| Recommendation to use partograph |  |  |
| Always | 181 | 91.4 |
| Some times | 17 | 8.6 |
| **Observations plot on the partograph** |  |  |
| Fetal heart Beat |  |  |
| Yes | 170 | 85.9 |
| No | 28 | 14.1 |
| Color of amniotic |  |  |
| Yes | 166 | 83.8 |
| No | 32 | 16.2 |
| Degree of molding |  |  |
| Yes | 167 | 84.3 |
| No | 31 | 15.7 |
| Dilatation of the cervix |  |  |
| Yes | 168 | 84.8 |
| No | 30 | 15.2 |
| Descent of the Head |  |  |
| Yes | 167 | 84.3 |
| No | 31 | 15.7 |
| Uterine contraction |  |  |
| Yes | 168 | 84.8 |
| No | 30 | 15.2 |
| Maternal blood pressure |  |  |
| Yes | 187 | 94.4 |
| No | 11 | 5.6 |
| Maternal Pulse rate |  |  |
| Yes | 188 | 94.9 |
| No | 10 | 5.1 |
| Maternal temperature |  |  |
| Yes | 189 | 95.5 |
| No | 9 | 4.5 |
| **Knowledge of mean score** |  |  |
| Satisfactory | 135 | 68.2 |
| Unsatisfactory | 63 | 31.8 |
|  |  |  |

**Table S2: Attitude of the participant towards the use of partograph among obstetric care givers at public health institutions in Central zone, Tigray, Ethiopia/2017**

| **Variables, N = 198** | **Frequency** | **Percentage** |
| --- | --- | --- |
| Partograph is an important tool to monitor labor |  |  |
| Agree | 198 | 100.0 |
| Disagree | 0 | 0 |
| Partograph should be used in all normal labor |  |  |
| Agree | 190 | 96.0 |
| Disagree | 8 | 4.0 |
| Partograph decreases mother and infant morbidity and mortality |  |  |
| Agree | 197 | 99.5 |
| Disagree | 1 | .5 |
| Partograph identify cases for surgery |  |  |
| Agree | 190 | 96.0 |
| Disagree | 8 | 4. |
| I want training on partograph use |  |  |
| Agree | 186 | 93.9 |
| Disagree | 12 | 6.1 |
| I wish to use partograph as a routine |  |  |
| Agree | 190 | 96.0 |
| Disagree | 8 | 4.0 |
| Partograph is responsibility of midwives only |  |  |
| Agree | 149 | 75.3 |
| Disagree | 49 | 24.7 |
| Training on partograph for nurse isn’t necessary |  |  |
| Agree | 178 | 89.9 |
| Disagree | 20 | 10.1 |
| Partograph isn’t effective in assessment of parturient |  |  |
| Agree | 163 | 82.3 |
| Disagree | 35 | 17.7 |
| Partograph is a loss of time |  |  |
| Agree | 157 | 79.3 |
| Disagree | 41 | 20.7 |
| I have difficulties in using partograph |  |  |
| Agree | 157 | 79.3 |
| Disagree | 41 | 20.7 |
| **Attitude of mean score** |  |  |
| Favorable Attitude | 134 | 67.7 |
| Unfavorable attitude | 64 | 32.3 |
